# 2-Phenylnaphthalene Derivatives Inhibit Lipopolysaccharide-Induced Pro-Inflammatory Mediators by Downregulating of MAPK/NF-κB Pathways in RAW 264.7 Macrophage Cells

**DOI:** 10.1371/journal.pone.0168945

**Published:** 2017-01-06

**Authors:** Chi-Fen Chang, Kang-Chun Liao, Chung-Hwan Chen

**Affiliations:** 1 Department of Anatomy, School of Medicine, China Medical University, Taichung, Taiwan; 2 Department of Biomedical Imaging and Radiological Science, China Medical University, Taichung, Taiwan; 3 Department of Orthopedics, Kaohsiung Municipal Ta-Tung Hospital, Kaohsiung Medical University, Kaohsiung, Taiwan; 4 Department of Orthopedics, College of Medicine, Kaohsiung Medical University, Kaohsiung, Taiwan; 5 Orthopaedic Research Center, Kaohsiung Medical University, Kaohsiung, Taiwan; National Institute of Allergy and Infectious Diseases, UNITED STATES

## Abstract

The anti-inflammatory pharmacological effect of eight 2-phenylnaphthalenes (**PNAP**-**1**−**PNAP**-**8**) on lipopolysaccharide (LPS)-induced RAW 264.7 (a mouse cell line) was investigated. Among them, 6,7-dihydroxy-2-(4′-hydroxyphenyl)naphthalene (**PNAP**-**6**) and 2-(4′-aminophenyl)-6,7-dimethoxynaphthalene (**PNAP**-**8**) exhibited the best anti-inflammatory activity in this study. **PNAP**-**6** and **PNAP**-**8** not only significantly decreased the expression of inducible nitric oxide synthase and cyclooxygenase-II, but also inhibited the production of nitric oxide, interleukin-6, and tumor necrosis factor-α in LPS stimulated cells. Moreover, **PNAP**-**6** and **PNAP**-**8** inhibited nuclear factor (NF)-κB activation by decreasing the degradation of IκB and nuclear translocation of NF-κB subunit (p65). In addition, **PNAP**-**6** and **PNAP**-**8** also attenuated the phosphorylation of ERK, p38, and JNK. These results suggest that **PNAP**-**6** and **PNAP**-**8** exert anti-inflammatory activities by down regulating NF-κB activation and the mitogen-activated protein kinase signaling pathway in LPS-stimulated Raw 264.7 cells. This is the first study demonstrating that PNAPs can inhibit LPS-induced pro-inflammatory mediators in macrophages cells.

## Introduction

Inflammation is a defense mechanism induced in the host in response to injury or infection [1]. The inflammatory response is characterized by redness, swelling, heat, and pain [2]. These symptoms present significant issues for managing many diseases such as arthritis, cancer, and diabetes [3]. Thus, inhibition and prevention of inflammatory processes are important research goals.

Macrophages play a crucial role in the innate and adaptive immune response to pathogens and are import mediators of the inflammatory process [4]. Moreover, the RAW 264.7, a mouse leukaemic monocyte macrophage cell line, has been widely used to evaluate the inflammatory effects of compounds [5]. Macrophages produce and release pro-inflammatory mediators such as nitric oxide (NO), prostaglandin E_2_ (PGE_2_), interleukin-6 (IL-6), and tumor necrosis factor-α (TNF-α) in response to lipopolysaccharide (LPS) treatment [6]. NO is synthesized from L-arginine by inducible NO synthase (iNOS). PGE_2_ is produced from the catalysis of arachidonic acid metabolites by cyclooxygenase-II (COX-II). These inflammatory cytokines or enzymes are frequently overexpressed in many inflammatory diseases such as rheumatoid arthritis, myocarditis, and colitis [7,8]. Therefore, anti-inflammatory compounds hold tremendous potential for advancing the treatment of inflammatory disorders.

The mitogen-activated protein kinase (MAPK) signaling pathway is involved in the LPS-induced pro-inflammatory response [9]. Three major subfamilies of MAPKs have been identified, including: extracellular signal-related kinase (ERK), p38, and c-Jun N-terminal kinase (JNK). It is well-known that the activation of ERK is associated with LPS-induced TNF-α production in macrophages [10,11]. In addition, p38 activation is involved the production of inflammatory mediators to initiate leucocyte recruitment and activation and p38 positively regulates the expression of numerous inflammation-related genes such as those coding for TNF-a, IL-6, and COX-II [12,13]. JNK can be induced by bacterial endotoxin, inflammatory cytokines, hypoxia, and UV radiation [14]. In addition, JNK regulates activator protein (AP)-1 transcription factor activity and stimulates the expression of pro-inflammatory mediators such as TNF-α and COX-II [15]. Therefore, inhibition of the MAPK pathway may contribute to anti-inflammatory activities.

NF-κB is a transcription factor that plays an important role in inflammation [16], and p65 is an NF-κB family member and its subunit. In unstimulated cells, NF-κB remains in the cytosol as an IκB-NF-κB complex. After stimulation with LPS, cytosolic IκB is phosphorylated by IKB kinase (IKK). This phosphorylation causes it to dissociate from the IκB-NF-κB complex, allowing NF-κB to translocate to the nucleus. NF-κB then combines with target DNA elements to activate transcription of proinflammatory genes such as iNOS, COX-II, TNF-α, and IL-6 [17]. Therefore, an inhibitor of NF-κB may be effective as an anti-inflammatory agent.

It is well known that naphthalene derivatives (e.g., 2-(3-(3,4-dimethylphenyl)-5-(naphthalen-2-yl)-4,5-dihydro-1*H*-pyrazol-1-yl)thiazol-4(5*H*)-one, etc.) display anti-tumor, anti-arrhythmia, and antioxidant activities [18]. Many anti-inflammatory clinical drugs such as propranolol, naphazoline, and nabumetone possess the bicyclic naphthalene skeleton [19]. Recently, we reported that **PNAP**-**6** exhibits potent anti-cancer activity [20]. However, it was unclear whether the MAPK and NF-κB pathways were activated in PNAP-mediated anti-inflammation. In this study, we assessed the anti-inflammatory effects of a series of PNAPs in RAW 264.7 cells, and elucidated the underlying mechanism of PNAP-induced anti-inflammation.

## Materials and Methods

### Chemicals and cell culture

**Fig 1:**
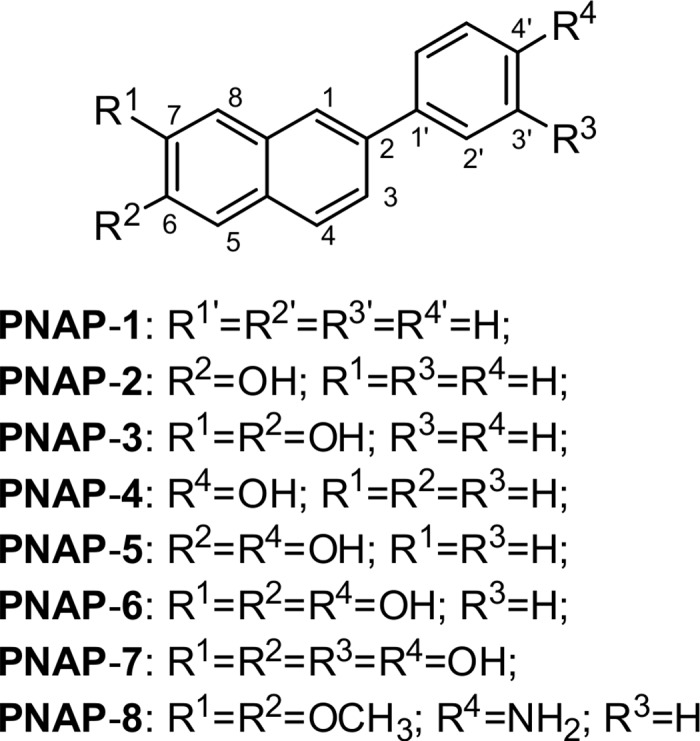
Chemical Structures of 2-Phenylnaphthalene Derivatives PNAP-1−PNAP-8.

2-Phenylnaphthalene (**PNAP**-**1**), 6-hydroxy-2-phenylnaphthalene (**PNAP-2**), 6,7-dihydroxy-2-phenylnaphthalene (**PNAP**-**3**), 2-(4′-hydroxyphenyl)naphthalene (**PNAP**-**4**), 6-hydroxy-2-(4′-hydroxyphenyl)naphthalene (**PNAP**-**5**), 6,7-dihydroxy-2-(4′-hydroxyphenyl)naphthalene (**PNAP**-**6**), 6,7-dihydroxy-2-(3′,4′-dihydroxyphenyl)naphthalene (**PNAP**-**7**), and 2-(4′-aminophenyl)-6,7-dimethoxynaphthalene (**PNAP**-**8**) were provided by Dr. Ta-Hsien Chuang (School of Pharmacy, China Medical University, Taiwan). Their molecular structures are shown in Fig 1. Griess reagent (sulfanilamide, phosphoric acid, and *N*-(1-naphthyl)ethylenediamine dihydrochloride), and primary antibody against proliferating cell nuclear antigen (PCNA) were purchased from Sigma-Aldrich (St. Louis, MO). These primary antibodies against iNOS, COX-II, phospho-ERK, phospho-p38, phospho-JNK, ERK, p38, JNK, IκBα, NF-κB subunit (p65), and an HRP-linked goat anti-rabbit IgG secondary antibody were purchased from Cell Signaling Technology (Beverly, MA). β-actin was purchased from Biovision (Mountain View, CA). JNK inhibitor (SP600125, 10 mM 2*H*-dibenzo[*cd*,*g*]indazol-6-one in dimethyl sulfoxide (DMSO), ERK inhibitor (PD98059, 10 mM 2-(2-amino-3-methoxyphenyl)chromen-4-one in DMSO), and p38 inhibitor (SB203580, 4-[4-(4-fluorophenyl)-2-(4-methylsulfinylphenyl)-1*H*-imidazol-5-yl]pyridine) were purchased from Selleckchem Inc. (Houston, TX, USA). RAW 264.7 cell lines were obtained from the laboratory of Dr. Yi-Ying Wu (Department of Medical Laboratory Science and Biotechnology, China Medical University, Taiwan). RAW 264.7 cells were maintained in DMEM/F12 supplemented with 10% heat-inactivated fetal bovine serum and 1% penicillin-streptomycin at 37°C in a humidified 5% CO_2_ incubator.

### Cell viability assay

RAW 264.7 cells were plated at a density of 1 × 10^4^ cells per well in 96-well plates, incubated overnight, and then treated with different concentrations (0, 0.25, 0.5, 1, 2.5, 5, 10, 25, and 50 μM) of **PNAP**-**1**−**PNAP**-**8**. After incubation, 10 μL of MTT solution (5 mg/mL in PBS) was added to each well, and the cells were incubated for an additional 1 h at 37°C. Subsequently, the medium was completely removed, and 50 μL of DMSO was added to solubilize the MTT formazan crystals. Finally, the absorbance was measured with an Epoch Microplate Reader (BioTek Instruments, VT, USA) at a wavelength of 550 nm. Cell viability (100%) = [OD of test samples (MTT plus PNAP plus medium with cells)—OD blank (MTT plus PNAP plus medium without cell)]/[OD of control samples (MTT plus medium with cell)] × 100%.

### Determination of iNOS, COX-II, MAPK expression

Cells were seeded at 8 × 10^5^/mL per well in a 6-well plate overnight. Cells were divided into four groups: 1) media-only; 2) PNAPs-treated with different concentrations; 3) LPS-only; and 4) PNAPs -pre-treated with different concentrations for 1 h and then stimulated with 0.1 μg/mL LPS for 15 min (for expression of phospho-p38, phospho-ERK, phospho-JNK, p38, ERK, JNK, NF-κB subunit (p65), and PCNA) or 24 h (for expression of iNOS and COX-II). Cells were collected and washed twice in PBS and then lysed in RIPA buffer for 30 min on ice. Lysates were centrifuged at 14,000 rpm for 30 min at 4°C. The protein concentration was measured using a Bio-Rad assay and an Epoch Microplate Reader (BioTek Instruments, VT, USA). Cell lysates were separated by 10% SDS-PAGE and electrophoretically transferred to polyvinylidene difluoride (PVDF) membranes (Millipore, Bedford, MA). After several washes, membranes were blocked with 5% skim milk in TBST for 1 h at room temperature and incubated with different primary antibodies (specific for iNOS, COX-II, phospho-ERK, phospho-p38, and phospho-JNK) at 4°C overnight. Membranes were then washed three times with TBST and probed with horseradish peroxidase (HRP)-conjugated secondary antibody for 1 h at room temperature. After washing three times in TBST, bound antibody was visualized using ECL Western Blotting Reagent (PerkinElmer, Boston), and the chemiluminescence was detected using Fuji Medical X-ray film (Tokyo, Japan).

### Determination of nitric oxide (NO) production

Cells were seeded at 1.5 × 10^5^/mL per well in a 24-well plate overnight. Cells were divided into four groups: 1) media-only; 2) PNAP-1−PNAP-8-treated with different concentrations; 3) LPS-only; and 4) PNAP-1−PNAP-8-pre-treated with different concentrations for 1 h and then stimulated with 0.1 μg/mL LPS for 24 h. The culture medium was then collected and centrifuged for 5 min at 1000 × *g*. After centrifugation, 100 μL of supernatant was transferred to a new 96-well plate, and an equivalent amount of Griess reagent was added. The optical density (OD) of the mixture was measured using an Epoch Microplate Reader (BioTek Instruments, VT, USA) at 540 nm.

### Detection of cytokine production

Cells were seeded at 1.5 × 10^5^/mL per well in a 24-well plate overnight. Cells were divided into four groups: 1) media-only; 2) PNAP-1−PNAP-8-treated with different concentrations; 3) LPS-only; and 4) PNAP-1−PNAP-8-pre-treated with different concentrations for 1 h and then stimulated with 0.1 μg/mL LPS for 24 h. Following stimulation, the culture medium was collected and centrifuged at 1000 × *g* for 5 min to obtain cell-free supernatant. Supernatants were stored at -80°C before being assessed for various cytokines. The concentrations of IL-6 and TNF-α in the supernatants were measured by commercial DuoSet ELISA kits (R&D Systems Europe, Abingdon, Oxon, UK) according to the manufacturer^’^s instructions. Both TNF-α and IL-6 were measured in triplicate, and the ELISA plates were read using an Epoch Microplate Reader (BioTek Instruments, VT, USA) at 450 nm.

### Determination of IκBα and NF-κB subunit (p65) expression

Cells were seeded at 2 × 10^6^/mL per well in a 6 cm dish overnight. Cells were divided into four groups: 1) media-only; 2) PNAPs-treated with different concentrations; 3) LPS-only; and 4) PNAPs -pre-treated with different concentrations for 1 h and then stimulated with 0.1 μg/mL LPS for 15 min. Cells were detached by gentle scraping and re-suspended in ice-cold PBS. Nuclear and cytoplasmic extracts were isolated using the NE–PER Nuclear and Cytoplasmic Extraction Reagents Kit (Pierce, Rockford, IL, USA) according to the manufacturer^’^s instructions. The extracts were stored at -80°C until further western blot analyses. The cytoplasmic extracts were probed with IκBα and NF-κB subunit (p65) antibodies. Nuclear extracts were probed with only the NF-κB subunit (p65) antibody.

### Statistical analysis

The results are presented as the mean ± SEM of three independent experiments performed in triplicate. Data were normalized to the level of the control in each sample. Differences between the control group and experimental groups were compared by analysis of variance (ANOVA) and the post hoc Tukey Honestly Significant Difference (HSD) test using SPSS 12.0 software for Windows (USA). P values < 0.05 were considered statistically significant.

## Results

### Effect of PNAPs on RAW 264.7 cell viability

**Fig 2:**
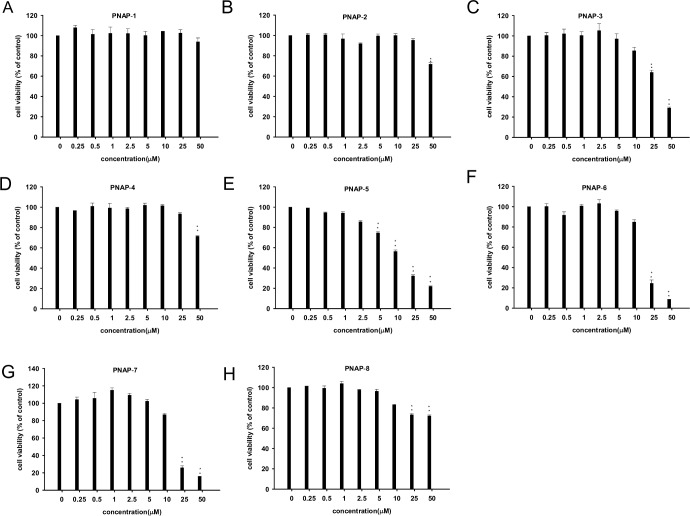
Effect of PNAPs on the viability of RAW 264.7 cells. Cells were incubated with PNAPs (0–50 μM) for 24 h. Cell viability was examined by MTT assays. (A) **PNAP**-**1**, (B) **PNAP**-**2**, (C) **PNAP**-**3**, (D) **PNAP**-**4**, (E) **PNAP**-**5**, (F) **PNAP**-**6**, (G) **PNAP**-**7**, and (H) **PNAP**-**8**. Data represent the mean ± SEM from three independent experiments. *P < 0.05 and **P < 0.01 compared to the control.

To determine the maximally effective concentration of **PNAP**-**1**−**PNAP**-**8** with minimal cytotoxicity and a viability percentage of more than 85%, RAW 264.7 cells were treated with the indicated concentrations of **PNAP**-**1**−**PNAP**-**8** for 24 h. RAW 264.7 cell viability, as determined by MTT assays, is shown in Fig 2. Concentrations of **PNAP**-**1** ≤ 50 μM, **PNAP**-**2** and **PNAP**-**4** ≤ 25 μM, **PNAP**-**3**, **PNAP**-**6**, **PNAP**-**7**, and **PNAP**-**8** ≤ 10 μM, and **PNAP**-**5** ≤ 2.5 μM treatment did not significantly influence cell viability. Thus, these concentrations were used in subsequent experiments.

### Effects of PNAPs on iNOS and COX-II expression in LPS-stimulated RAW 264.7 cells

**Fig 3:**
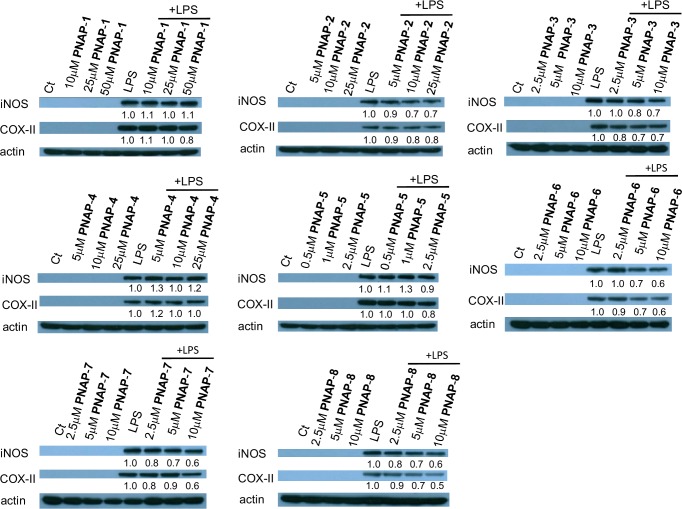
Effects of PNAPs on iNOS and COX-II expression in LPS-stimulated RAW 264.7 cells. Cells were pre-treated with PNAPs at different concentrations for 1 h and then stimulated with or without 0.1 μg/mL LPS for 24 h. iNOS and COX-II protein expression were detected by western blotting. Results were normalized to β-actin expression. The protein levels of iNOS and COX-II after LPS treatment is expressed as the control, which was arbitrarily assigned a value of 1.0.

A previous study also revealed that significant increases in the expression of iNOS and COX-II protein were observed in LPS-stimulated RAW264.7 cells [6]. Here, we pre-treated cells with different concentrations of PNAPs for 1 h, and then stimulated them with (or without) 0.1 μg/mL LPS for 24 h; the expression of iNOS and COX-II were measured by western blotting. Expression of iNOS and COX-II was detected only in LPS-stimulated cells and not in cells that were not exposed to LPS (Fig 3). LPS induced expression of iNOS and COX-II, which was inhibited by **PNAP**-**2**, -**3**, -**6**, -**7**, and -**8** pre-treatment. Among them, the pro-inflammatory mediators iNOS and COX-2 expression ratios of **PNAP**-**6**, -**7**, or -**8** plus LPS-treatment / only LPS-treatment were 40***−***50%.

### Effects of PNAPs on NO production in LPS-stimulated RAW 264.7 cells

**Fig 4:**
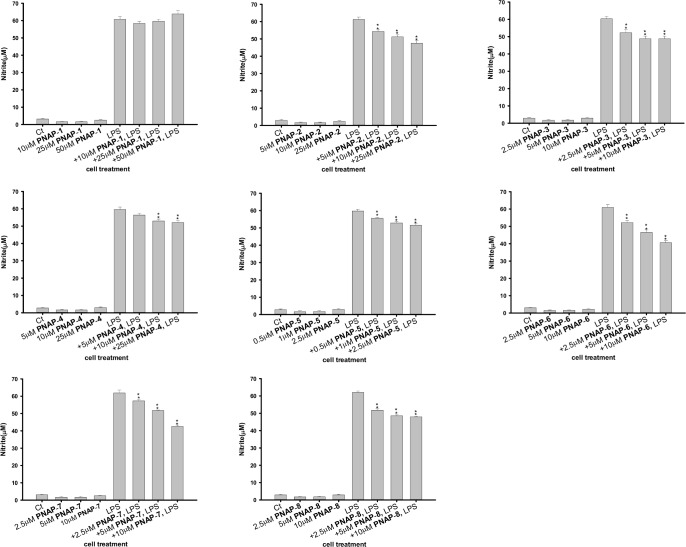
Effect of PNAPs on NO expression in LPS-stimulated RAW 264.7 cells. Cells were pre-treated with PNAPs in different concentrations and exposed to 0.1 μg/mL LPS for 24 h. NO production in the supernatant was measured using the Griess reaction. The + symbols on the x-axis indicate pre-treatment with PNAPs. Data represent the mean ± SEM from three independent experiments. *p < 0.05, **p < 0.01, indicate significant differences compared to the LPS-treated group.

The free NO radical, produced by iNOS, is involved in the regulation of inflammatory responses [6]. We investigated whether **PNAP**-**1**−**PNAP**-**8** modulated NO formation in LPS-stimulated RAW 264.7 cells by NO production, which was quantified using the Griess assay (see Materials and Methods: Determination of NO production). As shown in Fig 4, a low level of NO production was observed in the cells after treatment with **PNAP**-**1**−**PNAP**-**8** without LPS. A dramatic increase in NO production was observed in cells following LPS stimulation for 24 h. However, this increase was significantly suppressed by pre-treatment with various concentrations of **PNAP**-**2**−**PNAP**-**8**. Among them, the pro-inflammatory mediator NO expression ratios of **PNAP**-**6**, -**7**, or -**8** plus LPS-treatment / only LPS-treatment were approximately 40%.

### Effects of PNAPs on IL-6 and TNF-α production in LPS-stimulated RAW 264.7 cells

**Fig 5:**
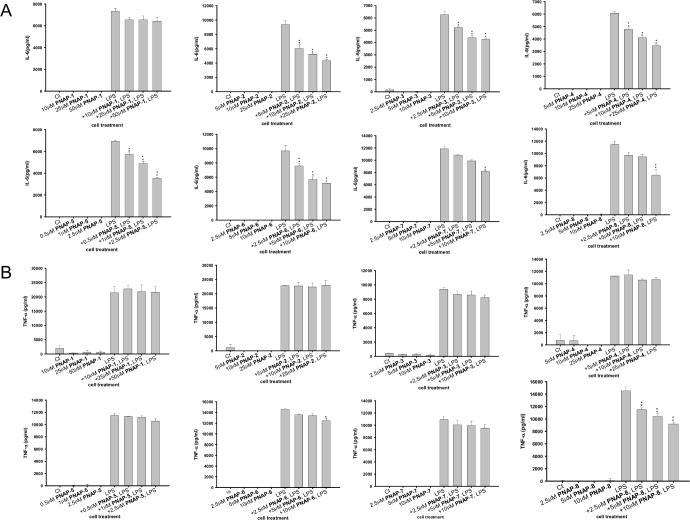
Effects of PNAPs on IL-6 and TNF-α expression in LPS-stimulated RAW 264.7 cells. After pre-treatment with PNAPs at different concentrations for 1 h, cells were stimulated with or without LPS (0.1 μg/mL) for 24 h. The concentration of (A) IL-6 or (B) TNF-α in medium was measured by ELISA. The + symbols on the x-axis indicate pre-treatment with PNAPs. Data are expressed as the means ± SEM from three independent experiments. *p < 0.05 or **p < 0.01 compared to the LPS group. Note: in panel A and B, for cells treated with PNAP only, IL-6 and TNF-α concentrations were low or undetectable using the IL-6 and TNF-α ELISA kit.

Because LPS could induce pro-inflammatory cytokine production, we evaluated the anti-inflammatory effects of PNAPs on cytokine (IL-6 and TNF-α) production in LPS-stimulated macrophages using ELISA assays. As shown in Fig 5A and 5B, IL-6 and TNF-α expression was negligible or undetectable in cells exposed to different concentrations of **PNAP**-**1**−**PNAP**-**8** without LPS stimulation. **PNAP**-**1** pre-treatment in RAW 264.7 cells slightly decreased LPS-induced IL-6 production, but it was not statistically significant. However, **PNAP**-**2**−**PNAP**-**8** pre-treatment significantly decreased LPS-induced IL-6 production in RAW 264.7 cells (Fig 5A). Among them, the pro-inflammatory mediator IL-6 production ratios of **PNAP**-**6** or **PNAP**-**8** plus LPS-treatment / only LPS-treatment were approximately 50%. In addition, LPS-induced TNF-α production was downregulated by **PNAP**-**8** in a dose-dependent manner (Fig 5B).

### Effects of PNAPs on MAPK and NF-κB activation in LPS stimulated RAW 264.7 cells

**Fig 6:**
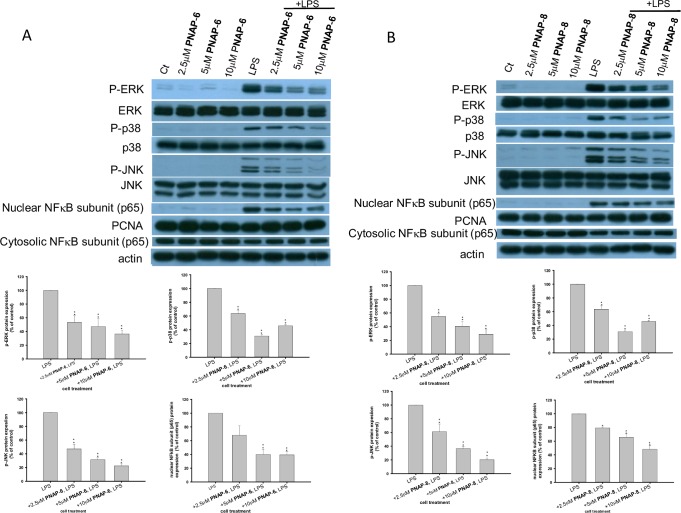
Effects of PNAPs on MAPK and NF-κB activation in LPS-stimulated RAW 264.7 cells. Cells were pre-treated with (A) **PNAP**-**6** or (B) **PNAP**-**8** (2.5, 5, and 10 μM) and exposed to 0.1 μg/mL LPS for 15 min. phospho-ERK, phospho-p38, phospho-JNK, ERK, p38, JNK, IκBα, and NF-κB subunit (p65), were then analyzed by western blotting. The + symbols on the x-axis indicate pre-treatment with PNAPs. The data are expressed as the means ± SEM from three independent experiments. *P < 0.05 and **P < 0.01 are significantly different from the control.

**Fig 7:**
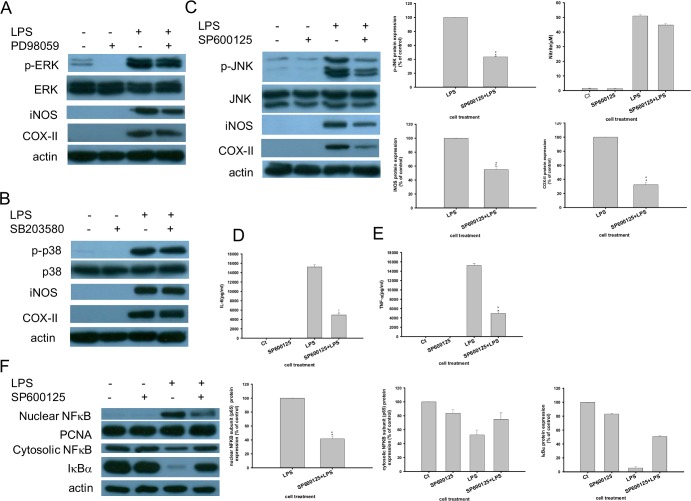
Effects of MAPK inhibitors on iNOS, COX-II, NF-κB, and IκBα expression in LPS-stimulated RAW 264.7 cells. Cells were pre-treated with (A) ERK inhibitor (PD98059), (B) p38 inhibitor (SB203580), or (C) JNK inhibitor (SP600125) for 1 h and stimulated with LPS (0.1 μg/mL) for 15 min (for evaluation of expression of phospho-ERK, phospho-p38, phospho-JNK, ERK, p38, and JNK) or 24 h (for expression of iNOS and COX-II). For data in (A), (B), and (C), phospho-JNK, phospho-ERK, phospho-p38, ERK, p38, JNK, iNOS, and COX-II levels were detected by western blot analysis. Cells were pre-treated with 10 μM JNK inhibitor (SP600125) for 1 h before treatment with or without LPS (0.1 μg/mL) for 24 h. (D) IL-6 and (E) TNF-α production were determined by an ELISA assay. (F) Cells were pre-treated with JNK inhibitor (SP600125) (10 μM) and exposed to 0.1 μg/mL LPS for 15 min. The expression of cytosolic NF-κB subunit (p65) and IκBα and nuclear NF-κB subunit (p65) was determined by western blot analysis. All data are expressed as the means ± SEM from three independent experiments. *P < 0.05 and **P < 0.01 are significantly different from the control.

On the basis of the effects of PNAPs on iNOS and COX-II expression (Fig 3), NO production (Fig 4), as well as IL-6 and TNF-α production (Fig 5), **PNAP**-**6** and **PNAP**-**8** were further evaluated to determine the underlying mechanism. LPS triggers a series of signal transduction events that lead to activation of MAPK signaling pathways [21]. To understand the molecular mechanism of PNAPs in the protection against LPS-induced activation in RAW 264.7 cells, the expression of phospho-ERK, phospho-p38, phospho-JNK, ERK, p38, and JNK was measured. First, western blot analysis showed that upon LPS stimulation, phospho-ERK, phospho-p38, and phospho-JNK levels were markedly elevated, but the levels of unphosphorylated (inactive) ERK, p38, and JNK proteins did not change. Upon treatment of cells with LPS and **PNAP**-**6** (or **PNAP**-**8**), the expression of phospho-ERK, phospho-p38, and phospho-JNK decreased in a dose-dependent manner, while the levels of the unphosphorylated ERK, p38, and JNK proteins did not change (Fig 6A and 6B). To further determine if inhibition of MAPK contributes to the inhibitory action of **PNAP**-**6** (or **PNAP**-**8**), cells were treated with LPS with or without a ERK inhibitor (PD98059), a p38 inhibitor (SB203580), or a JNK inhibitor (SP600125). The results showed that JNK inhibitor (SP600125) inhibited LPS-induced phospho-JNK expression (Fig 7C). In addition, LPS-induced increases in iNOS, COX-II, IL-6, and TNF-α were also inhibited by JNK inhibitor (SP600125) (Fig 7C–7E). However, LPS-induced increases in pro-inflammatory mediators, as well as phospho-ERK and phospho-p38 levels, did not inhibit p38 or ERK by the ERK inhibitor (PD98059) and p38 inhibitor (SB203580) in our experiments (Fig 7A and 7B). Next, we wanted to understand whether **PNAP**-**6** and **PNAP**-**8** could inhibit LPS-induced NF-κB activation. The results showed that pre-treatment with **PNAP**-**6** and **PNAP**-**8** significant reduced LPS-induced NF-κB subunit (p65) nuclear accumulation, compared to that with LPS treatment alone (Fig 6A and 6B). Similarly, increased cytosolic distribution of IκBα and NF-κB subunit (p65) were observed with **PNAP**-**6** and **PNAP**-**8** pre-treatment. We also investigated whether **PNAP**-**6** and **PNAP**-**8** could mediate NF-κB activation through the regulation of phospho-JNK. We pre-treated RAW 264.7 cells with JNK inhibitor (SP600125), and analyzed the NF-κB subunit (p65) protein levels in both the cytoplasm and the nucleus and the cytosolic levels of IκBα by western blotting. As shown in Fig 7F, JNK inhibitor (SP600125) reduced the LPS-induced increase of nuclear NF-κB subunit (p65) in RAW 264.7 cells. Consistent with the above results, the cytosolic levels of the NF-κB subunit (p65) and IκBα in LPS-stimulated cells pre-treated with JNK inhibitor (SP600125) were higher than in LPS-stimulated cells. The results suggest that JNK signaling mediates **PNAP**-**6**- and **PNAP**-**8**-induced NF-κB activation.

## Discussion

A tri-hydroxy-2-phenylnaphthalene (**PNAP**-**6**) was previously shown to exhibit significant anticancer activity in MCF-7 cells [20]. Inflammation is closely linked to cancer [22]. Therefore, in this study, we evaluated the effects of **PNAP**-**1**−**PNAP**-**8** on the pro-inflammatory mediator using mouse RAW 264.7 cells. The RAW 264.7 cell line is a good model for studying inflammatory responses because LPS can activate RAW264.7 cells and trigger the production of inflammatory mediators such as TNF-α, IL-6, iNOS, and COX-II [23]. The RAW264.7 cell line was also found to be uniquely efficient for measuring infection-related pro-inflammatory mediators [24,25].

First, MTT assays were performed to measure RAW 264.7 cell viability of **PNAP**-**1**−**PNAP**-**8** to ensure that any anti-inflammatory effects were not attributed to decreasing RAW 264.7 cell viability (Fig 2). Based on these results, we chose the concentrations of **PNAP**-**1**−**PNAP**-**8** showing minimal cytotoxicity with a viability percentage of more than 85% for subsequent experiments. In addition, pro-inflammatory mediators (such as the expression of iNOS and COX-II, production of NO, and release of IL-6 and TNF-α) were identified in RAW 264.7 cells following LPS-only stimulation.

In response to LPS, macrophages were activated and released pro-inflammatory mediators such as NO, iNOS, and COX-II as well as several cytokines (e.g., TNF-α and IL-6) [26]. Overexpression of either pro-inflammatory mediators or cytokines has been implicated in many inflammatory diseases such as rheumatoid arthritis, asthma, and myocarditis [3]. A previous study revealed that capillarisin, a flavonoid, inhibits iNOS and COX-II expression in LPS-induced RAW 264.7 macrophages [27]. Similarly, our results demonstrated that PNAPs pre-treatment significantly inhibited the overexpression of iNOS and COX-II and downregulated NO production in the supernatant of LPS-induced RAW 264.7 cells (Figs 3 and 4). The process of inflammation involves IL-6 and TNF-α [28]. During inflammatory processes, IL-6 stimulates the expression of monocyte chemoattractant protein-1, which in turn increases monocyte recruitment [29]. Furthermore, TNF-α enhances the recruitment of leukocytes to the site of inflammation [30]. Some studies have also shown that flavonoids (e.g., capillarisin, apigenin, and genistein) suppress pro-inflammatory cytokines such as TNF-α and IL-6 in LPS-stimulated RAW 264.7 cells [27,31,32]. Again, our results showed that PNAPs suppress the LPS-induced production of TNF-α and IL-6 in RAW 264.7 cells. Therefore, our findings suggest that PNAPs can inhibit inflammatory mediators by decreasing the expression of iNOS and COX-II and production of NO, IL-6, and TNF-α.

Transcription factors of the NF-κB family play critical roles in inflammation, immunity, and survival. Upon LPS stimulation, IKK phosphorylates IκBα, leading to nuclear translocation of NF-κB [6]. The suppression of NF-κB activation by capillarisin and genistein inhibits the increase in pro-inflammatory mediators such as iNOS, COX-II, TNF-α, and IL-6 in LPS-stimulated RAW264.7 cells [27,32]. In the present study, we showed that **PNAP**-**6** and **PNAP**-**8** pre-treatment in LPS-stimulated cells resulted in higher cytosolic levels of IκBα and NF-κB subunit (p65), thus blocking translocation of the NF-κB subunit (p65) to the nucleus (Fig 6A and 6B). This result indicates that **PNAP**-**6** and **PNAP**-**8** inhibited pro-inflammatory mediators by inhibiting NF-κB activation. In addition to NF-κB, MAPKs can be activated by many extracellular molecules to induce downstream phosphorylation of key signaling molecules related to inflammation [33]. The MAPK family plays important roles in LPS-induced pro-inflammatory cytokine production in many cell types [34]. In cells stimulated by LPS, the phosphorylation of MAPK is involved in the activation of transcription factors including AP-1, NF-κB, and subsequently produced cytokines [35,36]. Some reports revealed that pro-inflammatory mediators are regulated by the down-regulation of MAPK and NF-κB in LPS treated RAW 264.7 cells [37,38]. Consistently, our study showed that pre-treatment of RAW 264.7 cells with **PNAP**-**6** and **PNAP**-**8** decreased the LPS-induced activation of ERK, p38 MAPK, and JNK. However, the levels of MAPK proteins did not change, indicating that **PNAP**-**6** and **PNAP**-**8** blocked the activation of MAPKs, but not their biosynthesis (Fig 6A and 6B). In addition, the JNK inhibitor (SP600125) significantly reduced LPS-induced expression of phospho-JNK, IL-6, TNF-α, iNOS, COX-II, and NF-κB (Fig 7C–7E). PNAPs may inhibit pro-inflammatory mediators by down regulating the LPS-induced MAPK and NF-κB signaling pathways.

In this study, some PNAPs inhibited pro-inflammation mediators in the MAPK/NF-kB regulated pathway in LPS-induced RAW264.7 cells. Previous studies suggested that inhibitors of MAPK or NF-κB had a clinical benefit in asthma and rheumatoid arthritis [39,40]. However, further studies are needed to provide insight into the inhibitory effects of PNAPs and their clinical use, as this study was limited to macrophage response analysis.

## Conclusion

In summary, a series of hydrophilic PNAPs were evaluated for their anti-inflammatory effect in LPS-induced RAW 264.7 cells. **PNAP**-**6**, a 6,7-dihydroxy-2-phenylnaphthalene with a 4-hydroxyphenyl group, and **PNAP**-**8**, a 6,7-dimethoxy-2-phenylnaphthalene with one para-amino group on the phenyl ring, exhibited the strongest anti-inflammatory activity in our tests. In addition, PNAPs also inhibited LPS-induced activation of the MAPK pathway, NF-κB translocation, and IκBα degradation. Thus, **PNAP**-**6** and **PNAP**-**8** have inflammatory preventive properties and might be useful anti-inflammatory agents.
